# Identification of Cell Subpopulations and Interactive Signaling Pathways From a Single-Cell RNA Sequencing Dataset in Osteosarcoma: A Comprehensive Bioinformatics Analysis

**DOI:** 10.3389/fonc.2022.853979

**Published:** 2022-04-20

**Authors:** Rong Wu, Xiaojie Dou, Haidong Li, Zhenguo Sun, Heng Li, Yuxin Shen, Wei Weng, Jikang Min

**Affiliations:** Department of Orthopaedics, The First People’s Hospital of Huzhou, The First Affiliated Hospital of Huzhou University, Huzhou, China

**Keywords:** osteosarcoma, scRNA sequencing, cell types, interaction, NF-κB

## Abstract

Osteosarcoma is a type of highly aggressive bone tumor arising from primitive cells of mesenchymal origin in adults and is associated with a high rate of tumor relapse. However, there is an urgent need to clarify the molecular mechanisms underlying osteosarcoma development. The present study performed integrated bioinformatics analysis in a single-cell RNA sequencing dataset and explored the potential interactive signaling pathways associated with osteosarcoma development. Single-cell transcriptomic analysis of osteosarcoma tissues was performed by using the Seurat R package, the Gene Ontology (GO) and Kyoto Encyclopedia of Genes and Genomes (KEGG) pathway enrichment analysis of differentially expressed genes was performed by using the clusterProfiler R package, and the cell–cell interaction analysis was performed by using the CellPhoneDB package. Our results showed that 11 clustered cell types were identified across 11 osteosarcoma tissues, with cell types including “osteoblastic”, “myeloid”, “osteoblastic_proli”, “osteoclast”, and “tumor-infiltrating lymphocytes (TILs)” as the main types. The DEGs between different cell types from primary, metastatic, and recurrent osteosarcomas were mainly enriched in the GO terms including “negative regulation of hydrolase activity”, “regulation of peptidase activity”, “regulation of binding”, “negative regulation of proteolysis”, and “negative regulation of peptidase activity” and in the KEGG pathways including “transcriptional misregulation in cancer”, “cellular senescence”, “apoptosis”, “FoxO signaling pathway”, “cell cycle”, “NF-kappa B signaling pathway”, “p53 signaling pathway”, “pentose phosphate pathway”, and “protein export”. For the cell–cell communication network analysis, the different interaction profiles between cell types were detected among primary, metastatic, and recurrent osteosarcomas. Further exploration of the KEGG pathway revealed that these ligand/receptor interactions may be associated with the NF-κB signaling pathway and its interacted mediators. In conclusion, the present study for the first time explored the scRNA-seq dataset in osteosarcoma, and our results revealed the 11 clustered cell types and demonstrated the novel cell–cell interactions among different cell types in primary, metastatic, and recurrent osteosarcomas. The NF-κB signaling pathway may play a key role in regulating the TME of osteosarcoma. The present study may provide new insights into understanding the molecular mechanisms of osteosarcoma pathophysiology.

## Introduction

Osteosarcoma is a type of highly aggressive bone tumor arising from primitive cells of mesenchymal origin in adults and is associated with a high rate of tumor relapse (1, 2). The main treatments for this malignancy are surgical resection combined with multiagent chemotherapy. Unfortunately, the incidence of osteosarcoma is around 5/1,000,000, and patients with osteosarcoma had a 5-year overall survival rate of ~15% (3–5). To our best knowledge, the pathophysiology of osteosarcoma remains unclear. The pathogenesis of osteosarcoma is complicated by the tumor microenvironment (TME) including immune cells, malignant mesenchymal tumor cells, vascular networks, and fibroblasts (6, 7). Therefore, it is of great importance to examine the molecular mechanisms underlying the pathophysiology of osteosarcoma and develop novel therapies for the treatment of osteosarcoma.

The conventional transcriptomic profiling is performed on mixed cell populations, which has an insufficient resolution for detecting specific cellular types and fails to assess the complexity of intratumoral heterogeneity (8, 9). Up to date, single-cell RNA sequencing (scRNA-seq) plays an important role in identifying the intratumor heterogeneity of different types of cancers and the cellular interaction within the TME. Davidson et al. performed scRNA-seq to examine the stromal compartment in murine melanoma and draining lymph nodes at points across tumor development and revealed a dynamic stromal niche that promoted tumor growth (10). Maynard et al. demonstrated that biological features revealed by scRNA-seq were biomarkers of clinical outcomes in human lung cancer, which highlighted how therapy-induced adaptation of the multicellular ecosystem of metastatic cancer shaped clinical outcomes (11). Chung et al. performed scRNA-seq and demonstrated that breast cancer transcriptome exhibited a wide range of intratumoral heterogeneity, which was shaped by the tumor cells and immune cells in the surrounding microenvironment (12). To our best knowledge, various studies have performed scRNA-seq in osteosarcoma under different experimental settings. For example, Liu et al. showed that single-cell transcriptomics elucidated the complexity of the tumor microenvironment of treatment-naive osteosarcoma (13). Zhou et al. identified 11 major cell clusters based on unbiased clustering of gene expression profiles and canonical markers *via* RN sequencing of 100,987 individual cells from 7 primary, 2 recurrent, and 2 lung metastatic osteosarcoma lesions (14). These studies highlighted the important applications of scRNA-seq in deciphering the molecular mechanisms underlying osteosarcoma pathophysiology.

Recently, reanalysis of high-throughput datasets including the scRNA-seq data in cancer studies has revealed many important findings. In the present study, we further explored the scRNA-seq data from the GEO database (GSE152048) and investigated the novel signaling pathways that may contribute to osteosarcoma metastasis.

## Materials and Methods

### Data Source Collection

The scRNA-seq files were accessed from GSE152048 *via* the GEO database. The dataset was based on the 10X Genomics platform. The dataset includes 11 tumor samples from 11 osteosarcoma patients. Among them, eight lesions were osteoblastic osteosarcoma, consisting of six primary, one recurrent, and one lung metastatic lesions, and three were chondroblastic osteosarcoma, each being derived from primary, recurrent, and lung metastasis sites.

### Analysis of scRNA-seq Data

The scRNA-seq data were processed by using the Seurat R package according to standard protocols (15). We excluded cells with less than 200 detected genes and also genes that were detected in less than 3 cells and limited the mitochondria proportion to less than 20%. The data normalization was performed by using the LogNormalize method. T-distributed stochastic neighbor embedding (t-SNE), a non-linear dimensionality reduction method, was applied after principal component analysis (PCA) for unsupervised clustering and unbiased visualizing of cell populations on a two-dimensional map. The marker genes of each cluster were detected using the “FindAllMarkers” function, and the criteria for identifying marker genes were set as follows: absolute log2 fold change (FC) ≥1 and the minimum cell population fraction in either of the two populations was 0.25. The expression pattern of each marker gene among clusters was visualized by applying the “DotPlot” function in Seurat. Marker-based cell-type annotation was performed by using the SingleR package.

### Gene Ontology and the Kyoto Encyclopedia of Genes and Genomes Analyses

The clusterProfiler R package was used to perform the Gene Ontology (GO) and Kyoto Encyclopedia of Genes and Genomes (KEGG) pathway functional enrichment analysis. The marker genes were assigned to various biological processes (BPs), cellular components (CCs), molecular functions (MFs), and pathways. Significant enrichment was set as *P <*0.05.

### Cell–Cell Interaction Prediction in Single-Cell Transcriptomics Data

Cell–cell interactions among all cell types were predicted based on the single-cell RNA sequencing data with the CellPhoneDB package (version 2.0.0) (16). The mean of the individual partner average expression values in the corresponding interacting pairs between different cell types was compared, and only the ligand–receptor interaction with *P*-value <0.05 was used to predict cell–cell interaction in the cell types. All the R codes for plotting the figures are presented in the **Supplemental Materials**.

## Results

### Single-Cell Transcriptomic Analysis of Osteosarcoma Tissues

**Figure 1:**
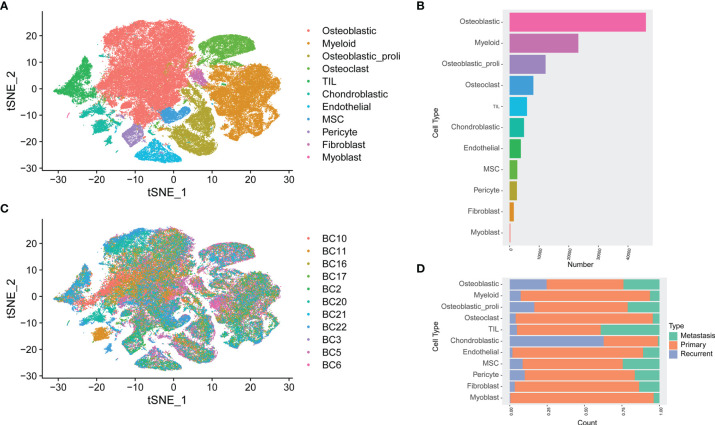
Single-cell transcriptomic analysis of osteosarcoma tissues. **(A)** The t-SNE plot of different cell types in osteosarcoma tissues. **(B)** The relative proportion of each cell cluster in all osteosarcoma tissues was shown. **(C)** Similar t-SNE plot of different cell types colored according to the individual osteosarcoma sample. **(D)** The relative proportion of each cell cluster according to different types of osteosarcoma was shown.

Based on the analysis of GSE152048, unbiased clustering of the cells identified 11 main clusters in parallel. The t-SNE plot of the different cell types based on the gene profiles and canonical markers in osteosarcoma tissues is shown in **Figure 1A**. As shown in **Figure 1B**, the relative proportion of each cell type in all osteosarcoma tissues was shown, and the osteoblastic cells were the largest population among all the cell types. Furthermore, the t-SNE plot of different cell types colored according to the individual osteosarcoma sample was shown (**Figure 1C**). The relative proportion of each cell cluster according to different types of osteosarcoma is shown in **Figure 1D**, and all the cell types except chondroblastic cells were found in primary osteosarcoma, while a large proportion of chondroblastic cells was detected in recurrent osteosarcoma.

### Functional Enrichment Analysis of Differentially Expressed Genes

**Figure 2:**
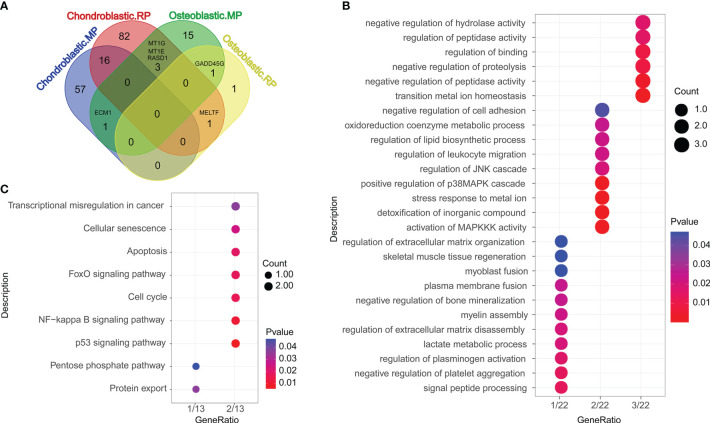
Functional enrichment analysis of differentially expressed genes (DEGs). **(A)** The Venn diagram showed the overlapped DEGs according to different cell types. Chondroblastic. MP = the DEGs in chondroblastic cells from metastatic osteosarcoma as compared with those from primary osteosarcoma. Chondroblastic. RP = the DEGs in chondroblastic cells from recurrent osteosarcoma as compared with those from primary osteosarcoma. Osteoblastic. MP = the DEGs in osteoblastic cells from metastatic osteosarcoma as compared with those from primary osteosarcoma. Osteoblastic. RP = the DEGs in osteoblastic cells from recurrent osteosarcoma as compared with those from primary osteosarcoma. **(B)** The GO enrichment analysis of overlapped DEGs between each of two groups. **(C)** The KEGG pathway enrichment analysis of overlapped DEGs between each of two groups.

Differentially expressed genes (DEGs) compared between different groups are shown in **Figure 2A**, and the overlapped genes between different groups are illustrated in the Venn diagram (**Figure 2A**). A total of 16, 1, 3, 1, and 1 DEGs were, respectively, detected between the chondroblastic.MP and the chondroblastic.RP groups, the chondroblastic.MP and the osteoblastic.MP groups, the chondroblastic.RP and the osteoblastic.MP groups, the chondroblastic.RP and the osteoblastic.RP groups, and the osteoblastic.MP and the osteoblastic.RP groups. The 22 overlapped DEGs were subjected to GO and KEGG pathway enrichment analysis. The DEGs were mainly enriched in the GO terms including “negative regulation of hydrolase activity”, “regulation of peptidase activity”, “regulation of binding”, “negative regulation of proteolysis”, “negative regulation of peptidase activity”, and so on (**Figure 2B**). The DEGs were mainly enriched in the KEGG pathways including “transcriptional misregulation in cancer”, “cellular senescence”, “apoptosis”, “FoxO signaling pathway”, “cell cycle”, “NF-kappa B signaling pathway”, “p53 signaling pathway”, “pentose phosphate pathway”, and “protein export” (**Figure 2C**).

### Cell–Cell Communication Network Among Different Cell Types in Primary, Metastatic, and Recurrent Osteosarcomas

**Figure 3:**
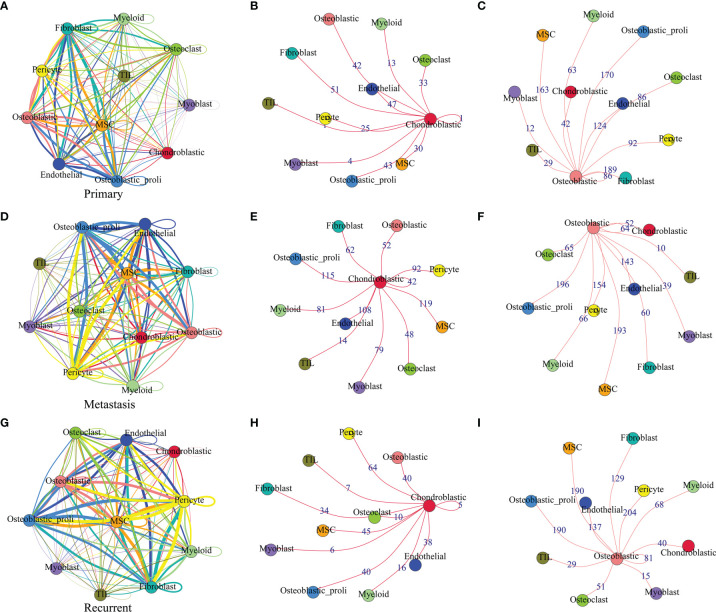
Cell–cell communication network among different cell types in primary, metastatic, and recurrent osteosarcomas. **(A)** The cell–cell communication network among different cell types in primary osteosarcoma. **(B)** The communication network between chondroblastic cells and other types of cells in primary osteosarcoma. **(C)** The communication network between osteoblastic cells and other types of cells in primary osteosarcoma. **(D)** The cell–cell communication network among different cell types in metastatic osteosarcoma. **(E)** The communication network between chondroblastic cells and other types of cells in metastatic osteosarcoma. **(F)** The communication network between osteoblastic cells and other types of cells in metastatic osteosarcoma. The thickness of the line or the number on the line is proportional to the number of ligand–receptor pairs connecting the two cell types. **(G)** The cell–cell communication network among different cell types in recurrent osteosarcoma. **(H)** The communication network between chondroblastic cells and other types of cells in recurrent osteosarcoma. **(I)** The communication network between osteoblastic cells and other types of cells in recurrent osteosarcoma.

The cell–cell communication network among different cell types in primary osteosarcoma is shown in **Figure 3A**. The detailed analysis revealed that chondroblastic cells mainly interacted with cell types including “fibroblast”, “osteoblastic”, “osteoblastic_proli”, “osteoclast”, “MSC”, and so on (**Figure 3B**); osteoblastic cells mainly interacted with cell types including “osteoblastic_proli”, “fibroblast”, “endothelial”, “MSC”, “pericyte”, “osteoclast”, and so on (**Figure 3C**). The cell–cell communication network for different types of cells in metastatic osteosarcoma is illustrated in **Figure 3D**. Further detailed analysis showed that chondroblastic cells mainly interacted with cell types including “osteoblastic_proli”, “endothelial”, “MSC”, “pericyte”, “myoblast”, “myeloid”, and so on (**Figure 3E**); osteoblastic cells mainly interacted with cell types including “osteoblastic_proli”, “MSC”, “endothelial”, “pericyte”, and so on (**Figure 3F**). The cell–cell communication network for different types of cells in recurrent osteosarcoma is shown in **Figure 3G**. The detailed analysis showed that chondroblastic cells mainly interacted with cell types including “pericyte”, “MSC”, “osteoblastic_proli”, “osteoblastic”, “endothelial”, and so on (**Figure 3H**); osteoblastic cells mainly interacted with cell types including “osteoblastic_proli”, “pericyte”, “MSC”, “endothelial”, “chondroblastic”, and so on (**Figure 3I**).

### Ligand–Receptor Interactions Between Various Types of Cells From Primary, Metastatic, and Recurrent Osteosarcomas

**Figure 4:**
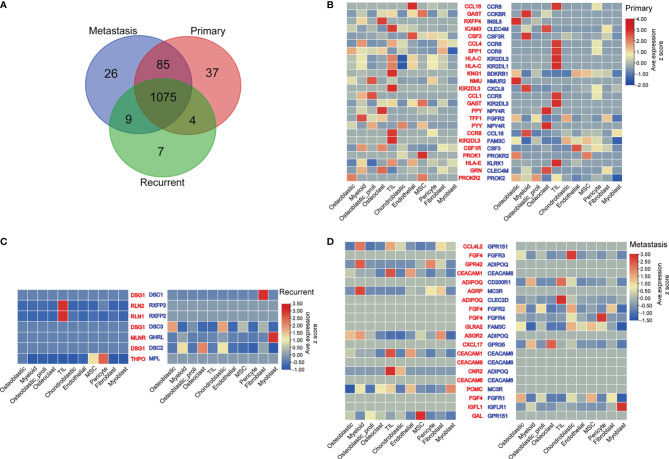
Ligand–receptor interactions between various types of cells from primary, metastatic, and recurrent osteosarcomas. **(A)** The common ligands/receptors in primary, metastatic, and recurrent osteosarcomas were illustrated as a Venn diagram. **(B)** The expression levels of ligands/receptors in all types of cells from primary osteosarcoma were shown as a heatmap plot. **(C)** The expression levels of ligands/receptors in all types of cells from metastatic osteosarcoma were shown as a heatmap plot. **(D)** The expression levels of ligands/receptors in all types of cells from recurrent osteosarcoma were shown as a heatmap plot.

The common ligands/receptors in all types of cells among primary, metastatic, and recurrent osteosarcomas are shown in **Figure 4A**, and a total of 1,075 common ligands/receptors were detected among primary, metastatic, and recurrent osteosarcomas. In addition, the expression of ligands/receptors in different types of cells in primary, metastatic, and recurrent osteosarcomas was illustrated as a heatmap plot (**Figures 4B–D**).

### Functional Enrichment Analysis and Interaction Between Nuclear Factor-κB Signaling Pathway and Ligands/Receptors

**Figure 5:**
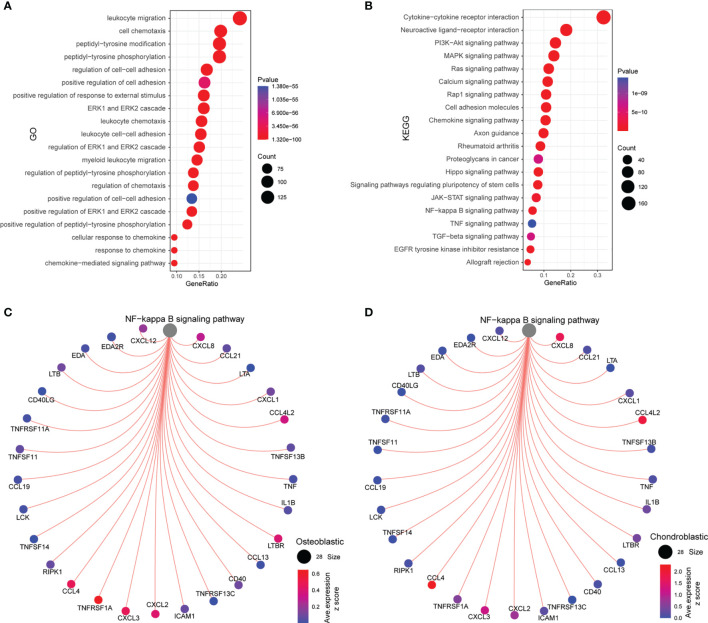
Functional enrichment analysis and interaction between the NF-κB signaling pathway and ligands/receptors. **(A)** GO enrichment analysis of common ligands/receptors among primary, metastatic, and recurrent osteosarcomas; **(B)** KEGG enrichment analysis of common ligands/receptors among primary, metastatic, and recurrent osteosarcomas. **(C)** The interaction between the NF-κB signaling pathway and common ligands/receptors in osteoblastic cells. **(D)** The interaction between the NF-κB signaling pathway and common ligands/receptors in chondroblastic cells.

As shown in **Figure 5A**, the common ligands/receptors were significantly enriched in the GO terms including “leukocyte migration”, “cell chemotaxis”, “peptidyl-tyrosine modification”, “peptidyl-tyrosine phosphorylation”, “regulation of cell–cell adhesion”, “positive regulation of cell adhesion”, and so on. For the KEGG pathway, the common ligands/receptors were significantly enriched in “cytokine–cytokine receptor interaction”, “neuroactive ligand–receptor interaction”, “PI3K–Akt signaling pathway”, “MAPK signaling pathway”, “NF-κB signaling pathway”, and so on (**Figure 5B**).

For the interaction between nuclear factor-κB (NF-κB) signaling pathway and common ligands/receptors in osteoblastic cells, the NF-κB signaling pathway showed a strong correlation with CCL4, TNFRSF1A, CXCL3, CXCL2, LTBR, CCL4L2, CXCL8, and CXCL12 (**Figure 5C**). For the interaction between NF-κB signaling pathway and common ligands/receptors in chondroblastic cells, the NF-κB signaling pathway showed a strong correlation with CXCL8, CCL4L2, CXCL2, CXCL3, and CCL4 (**Figure 5D**).

## Discussion

Accumulating evidence has implied that the biological behaviors of tumor cells are heavily affected by the tumor microenvironment. Network interactions among various types of tumor cells and the TME have been shown to promote tumor progression at multiple levels (1, 7, 17). Therefore, further understanding the underlying interactions should give rise to a novel therapeutic approach. Our results showed that 11 clustered cell types were identified across 11 osteosarcoma tissues, with cell types including “osteoblastic”, “myeloid”, “osteoblastic_proli”, “osteoclast”, and “tumor-infiltrating lymphocytes (TILs)” being the main types. The DEGs between different cell types from primary, metastatic, and recurrent osteosarcomas were mainly enriched in the GO terms including “negative regulation of hydrolase activity”, “regulation of peptidase activity”, “regulation of binding”, “negative regulation of proteolysis”, and “negative regulation of peptidase activity” and in the KEGG pathways including “transcriptional misregulation in cancer”, “cellular senescence”, “apoptosis”, “FoxO signaling pathway”, “cell cycle”, “NF-kappa B signaling pathway”, “p53 signaling pathway”, “pentose phosphate pathway”, and “protein export”. For the cell–cell communication network analysis, the different interaction profiles between cell types were detected among primary, metastatic, and recurrent osteosarcomas. Further exploration of the KEGG pathway revealed that these ligand/receptor interactions may be associated with the NF-κB signaling pathway and its interacted mediators. In conclusion, the present study for the first time explored the scRNA-seq dataset in osteosarcoma, and our results revealed the 11 clustered cell types and demonstrated the novel cell–cell interactions among different cell types in primary, metastatic, and recurrent osteosarcomas. The present study may provide new insights into understanding the molecular mechanisms of osteosarcoma pathophysiology.

Based on the study of Zhou et al., the researchers characterized the transcriptomic properties, regulators, and dynamics of osteosarcoma malignant cells together with their TME, particularly stromal and immune cells (14). In addition, the study revealed that the proinflammatory fatty acid-binding protein 4+ macrophage infiltration was identified in lung metastatic osteosarcoma lesions. Lower osteoclast infiltration was detected in chondroblastic, recurrent, and lung metastatic osteosarcoma lesions compared with primary osteoblastic osteosarcoma lesions. They found that TIGIT blockade enhanced the cytotoxicity effects of primary CD3+ T cells with a high proportion of TIGIT+ cells against osteosarcoma (14). In our analysis, the main cell types include “osteoblastic”, “myeloid”, “osteoblastic_proli”, and “osteoclast”. In metastatic osteosarcoma, there is a large proportion of TIL, MSC, and osteoblastic and mesenchymal stem cells (MSCs); in recurrent osteosarcoma, there is a large proportion of chondroblastic cells.

Several studies have proposed the importance of TIL in osteosarcoma. TILs were recruited to the tumor site; however, tumor cells had the capability of escaping the immune response (18). Studies found that the neutrophil–lymphocyte ratio was strongly correlated with the overall survival and progression-free survival of patients with osteosarcoma (18). Recent studies showed that higher infiltration of immune cells was correlated with better clinical outcomes in osteosarcoma (18). Sundara et al. showed that PD-L1 and T-cell infiltration were increased in the presence of HLA class I expression in metastatic high-grade osteosarcoma (19). The role of MSCs has also been reported in metastatic osteosarcoma. Tsukamoto et al. showed that mesenchymal stem cells promoted tumor engraftment and metastatic colonization in a rat osteosarcoma model (20); mechanistic studies showed that MSCs under stress increased osteosarcoma migration and apoptosis resistance *via* extracellular vesicle-mediated communication (21). Cortini et al. found that tumor-activated MSCs promoted osteosarcoma stemness and migratory potential *via* interleukin-6 secretion (22). Chondroblastic osteosarcoma ranks as the second commonly diagnosed osteosarcoma in young adults. The cellular origin of chondroblastic osteosarcoma is still unclear. In recurrent and metastatic osteosarcomas, the strong interaction between chondroblastic and osteoblastic cells was detected in our study, suggesting the transdifferentiation of malignant osteoblastic cells from malignant chondroblastic cells.

Based on the analysis of signaling pathways, we noticed that the NF-κB signaling pathway is key in the pathophysiology of osteosarcoma. NF-κB is involved in the early stress response to cellular DNA damage, and tumor necrosis factor α (TNFα) is an activator of the classical NF-κB pathway (23). After TNFα stimulation, IκB kinases (IKKs), such as IKKα and IKKβ, are first activated in cells. IKKs degrade intracellular IκB, thus releasing NF-κB molecules such as RelA, which are phosphorylated at S536, and this allows RelA to enter the nucleus (23), bind to genes at corresponding binding sites, and initiate the transcription of downstream genes, such as CCND1 and Bcl-2, to regulate cell cycle progression, proliferation, and survival (23). Nishimura et al. showed that transfection of NF-κB decoy oligodeoxynucleotide suppressed pulmonary metastasis by murine osteosarcoma (24). Londhe et al. showed that classical NF-κB metabolically reprogramed sarcoma cells through regulation of hexokinase 2 (25). Activation of the NF-κB axis could enhance CRL4B DCAF11 E3 ligase activity and regulate cell cycle progression in human osteosarcoma cells (26). Inhibition of NF-κB signaling could attenuate osteosarcoma progression (26, 27). In the present study, the costimulatory analysis revealed that ligands and receptors were correlated with NF-κB signaling. In future studies, we may perform functional studies to examine if disturbance of the ligands/receptors could lead to modulation of the NF-κB signaling pathway, which may eventually regulate the osteosarcoma progression.

The present was only focused on the bioinformatics analysis, which may limit the significance of the current findings. Thus, further studies especially validating the potential mediators detected in this study by using experimental assays should be considered. Due to the limited source of the scRNA-seq dataset for osteosarcoma, the present study only analyzed one dataset, and future studies should consider exploring other available scRNA-seq datasets to confirm the present findings. Moreover, our analysis only focused on the NF-κB signaling pathway and its interacted mediators in osteosarcoma, and other signaling pathways may be considered for exploration in our future studies.

## Conclusions

In conclusion, the present study for the first time explored the scRNA-seq dataset in osteosarcoma, and our results revealed the 11 clustered cell types and demonstrated the novel cell–cell interactions among different cell types in primary, metastatic, and recurrent osteosarcomas. The NF-κB signaling pathway may play a key role in regulating the TME of osteosarcoma. The present study may provide new insights into understanding the molecular mechanisms of osteosarcoma pathophysiology.

## Data Availability Statement

The original contributions presented in the study are included in the article/**Supplementary Material**. Further inquiries can be directed to the corresponding author.

## Author Contributions

JM and RW designed and supervised the whole project. RW, XD, HaL, ZS, and HeL processed the datasets and analyzed the data. JM wrote the manuscript. YS and WW revised the drafted manuscript. All authors contributed to the article and approved the submitted version.

## Funding

This study was supported by the Basic Public Welfare Research Program of Zhejiang (LGF19H060002).

## Conflict of Interest

The authors declare that the research was conducted in the absence of any commercial or financial relationships that could be construed as a potential conflict of interest.

## Publisher’s Note

All claims expressed in this article are solely those of the authors and do not necessarily represent those of their affiliated organizations, or those of the publisher, the editors and the reviewers. Any product that may be evaluated in this article, or claim that may be made by its manufacturer, is not guaranteed or endorsed by the publisher.
