# Supplementary material for: Identification of Cell Subpopulations and Interactive Signaling Pathways From a Single-Cell RNA Sequencing Dataset in Osteosarcoma: A Comprehensive Bioinformatics Analysis
Source: Front Oncol. 2022 Apr 20;12:853979. doi: 10.3389/fonc.2022.853979 (PMC9066489; doi:10.3389/fonc.2022.853979)
Supplement: Supplementary file 1 [file DataSheet_1.pdf]

R code for plotting Figure 1

```
library(Seurat)
OS <- readRDS("OS.combine.group.name.rds")
DimPlot(OS,reduction="tsne")
p1 <- DimPlot(OS,reduction="tsne")
p2 <- DimPlot(OS,reduction="tsne",group.by="orig.ident")
pdf("OS.group.sample.pdf")
CombinePlots(plots = list(p1, p2),ncol=1)
dev.off()
library(ggplot2)
library(Hmisc)
library(dplyr)
library(pheatmap)
library(reshape2)
Bcell <-OS
Bcell@meta.data$cellType<-Idents(OS)
ident_cluster <- Bcell@meta.data[,c('Type','seurat_clusters','cellType')]
num=length(levels(ident_cluster$cellType))
colours=colorRampPalette(c("#E41A1C","#377EB8","#4DAF4A","#984EA3","#FF7F00","#FFE528","#A65628","#F781BF","#999999"))(num)[num:1]
Sample_cluster_percent<-acast(ident_cluster ,Type~cellType,length)
ident_cluster$cellType <- factor(ident_cluster$cellType,levels=c("Myoblast","Fibroblast","Pericyte","MSC","Endothelial","Chondroblastic","TIL","Osteoclast","Osteoblastic_proli","Myeloid","Osteoblastic"))
ggplot(ident_cluster,aes(x=cellType,fill=Type)) + geom_bar(position='fill')+theme(axis.text.x=element_text(angle=90, size=8,colour="black",hjust=1,vjust=1))+coord_flip()+theme(panel.grid.major.x = element_blank(), panel.grid.minor.x = element_blank(),panel.grid.major.y = element_blank(),panel.grid.minor.y =element_blank())
```

R code for plotting Figure 2

```
# 安装包
```

```r
#source("https://bioconductor.org/biocLite.R")
#biocLite("DOSE")
#biocLite("topGO")
#biocLite("clusterProfiler")
#biocLite("pathview")

# 加载包
library(DOSE)
library(org.Hs.eg.db)
library(topGO)
library(clusterProfiler)
library(pathview)

# 导入数据
data <- read.table("gene.list",header=TRUE)
data$GeneName <- as.character(data$p_val)

# 转换基因名
transID = bitr(data$GeneName,fromType="SYMBOL",toType=c("ENSEMBL", "ENTREZID"),OrgDb="org.Hs.eg.db")

dir.create("GO")
dir.create("KEGG")

# GO_CC 注释
CC <- enrichGO(transID$ENTREZID,"org.Hs.eg.db",keyType="ENTREZID",ont="CC",pvalueCutoff=0.05,pAdjustMethod="BH",qvalueCutoff=0.1)
CC <- setReadable(CC, OrgDb=org.Hs.eg.db)

pdf(file="./GO/GO_CC.pdf", bg="transparent")
```

```
dotplot(CC, showCategory=30, color="pvalue", font.size=8, title="GO_CC") # + theme(axis.text.y = element_text(angle = 45))

barplot(CC, showCategory=30, title="GO_CC", font.size=8)

plotGOgraph(CC)

dev.off()


write.table(as.data.frame(CC@result), file="./GO/GO_CC.xls", sep="\t", row.names=F,quote=FALSE)


# GO_MF 注释

MF <- enrichGO(transID$ENTREZID, "org.Hs.eg.db", keyType="ENTREZID", ont="MF", pvalueCutoff=0.05, pAdjustMethod="BH", qvalueCutoff=0.1)

MF <- setReadable(MF, OrgDb=org.Hs.eg.db)


pdf(file="./GO/GO_MF.pdf", bg="transparent")

dotplot(MF, showCategory=30, color="pvalue", font.size=8, title="GO_MF") # + theme(axis.text.y = element_text(angle = 45))

barplot(MF, showCategory=30, title="GO_MF", font.size=8)

plotGOgraph(MF)

dev.off()


write.table(as.data.frame(MF@result), file="./GO/GO_MF.xls", sep="\t", row.names=F,quote=FALSE)


# GO_BP 注释

BP <- enrichGO(transID$ENTREZID, "org.Hs.eg.db", keyType="ENTREZID", ont="BP", pvalueCutoff=0.05, pAdjustMethod="BH", qvalueCutoff=0.1)

BP <- setReadable(BP, OrgDb=org.Hs.eg.db)


pdf(file="./GO/GO_BP.pdf", bg="transparent")

dotplot(BP, showCategory=30, color="pvalue", font.size=8, title="GO_BP") # + theme(axis.text.y = element_text(angle = 45))

barplot(BP, showCategory=30, title="GO_BP", font.size=8)
```

```
plotGOgraph(BP)
dev.off()

write.table(as.data.frame(BP@result), file="./GO/GO_BP.xls", sep="\t", row.names=F,quote=FALSE)

# KEGG 注释
kegg <- enrichKEGG(transID$ENTREZID, organism="hsa", pvalueCutoff=0.05, pAdjustMethod="BH", qvalueCutoff=0.1)
kegg <- setReadable(kegg, OrgDb=org.Hs.eg.db, keyType="ENTREZID")

pdf(file="./KEGG/KEGG.pdf", bg="transparent")
dotplot(kegg, showCategory=30, color="pvalue", font.size=8, title="KEGG") # + theme(axis.text.y = element_text(angle = 45))
barplot(kegg, showCategory=30, title="KEGG", font.size=8)
dev.off()

write.table(as.data.frame(kegg@result), file="./KEGG/kegg.xls", sep="\t", row.names=F,quote=FALSE)

#dir.create("./KEGG/MAP")
#kegg_df = as.data.frame(kegg)

#for(i in kegg_df$ID){
#	pathview(gene.data=transID$ENTREZID,pathway.id=i,species="hsa",kegg.native=TRUE,kegg.dir="./KEGG/MAP")
#}

print("TASK DONE")
```

R code for plotting Figure 3

```
rm(list = ls())
library(psych)
library(qgraph)
library(igraph)
library(tidyverse)
mynet <- read.table("Metastasis.count_network.txt",header=T,sep="\t")
#mynet %>% filter(count>0) -> mynet
mynet$SOURCE <- factor(mynet$SOURCE,levels=c("Chondroblastic","Endothelial","Fibroblast","MSC","Myoblast","Myeloid","Osteoblastic","Osteoblastic_proli","Osteoclast","Pericyte","TIL"))
mynet <- mynet[order(mynet$SOURCE),]
net<- graph_from_data_frame(mynet)
allcolour=c("#DC143C","#0000FF","#20B2AA","#FFA500","#9370DB",
        "#98FB98","#F08080","#1E90FF","#7CFC00","#FFFF00",
        "#808000","#FF00FF","#FA8072","#7B68EE","#9400D3",
        "#800080","#A0522D","#D2B48C","#D2691E","#87CEEB",
        "#40E0D0","#5F9EA0","#FF1493",
        "#FFE4B5","#8A2BE2","#228B22","#E9967A","#4682B4",
        "#32CD32","#F0E68C","#FFFFE0","#EE82EE","#FF6347",
        "#6A5ACD","#9932CC","#8B008B","#8B4513","#DEB887")
pdf("Single.Metastasis.comm.pdf")
for (i in 1: length(unique(mynet$SOURCE)) ){
    net1<-net
    E(net1)$count <- ""
    E(net1)[map(unique(mynet$SOURCE),function(x) {
    get.edge.ids(net,vp = c(unique(mynet$SOURCE)[i],x))
    })%>% unlist()]$count  <- E(net)[map(unique(mynet$SOURCE),function(x) {
    get.edge.ids(net,vp = c(unique(mynet$SOURCE)[i],x))
    })%>% unlist()]$count
    E(net1)[map(unique(mynet$SOURCE),function(x) {
```

```
    get.edge.ids(net,vp = c(unique(mynet$SOURCE)[i],x))})%>% unlist()]$color <-
allcolour[i]
    p<-plot(net1, edge.arrow.size=.1,
    edge.curved=0.4,
    edge.label = E(net1)$count,
    vertex.color=allcolour,
    vertex.frame.color="#555555",
    vertex.label.color="black",
    vertex.label.cex=1
    )
    print (p)
}
dev.off()

E(net)$width  <- E(net)$count/30
for (i in 1: length(unique(mynet$SOURCE)) ){
  E(net)[map(unique(mynet$SOURCE),function(x) {
  get.edge.ids(net,vp = c(unique(mynet$SOURCE)[i],x))
  })%>% unlist()]$color <- allcolour[i]
}
pdf("All.Metastasis.comm.pdf")
plot(net, edge.arrow.size=.1,
    edge.curved=0.2,
    vertex.color=allcolour,
    vertex.frame.color="#555555",
    vertex.label.color="black",
    vertex.label.cex=1)
dev.off()
```

R code for plotting Figure 4

```
library(psych)
```

```
library(qgraph)

library(igraph)

library(tidyverse)

#mypvals <- read.delim(paste0(pbmc,"pvalues.txt"), check.names = FALSE)

#mymeans <- read.delim(paste0(pbmc,"means.txt"), check.names = FALSE)


mypvals <- read.table("pvalues.txt",header=T,sep="\t")

mymeans <- read.table("means.txt",header=T,sep="\t")



chemokines <- grep("^CXC|CCL|CCR|CX3|XCL|XCR",
mymeans$interacting_pair,value = T)

th1 <- grep("IL2|IL12|IL18|IL27|IFNG|IL10|TNF$|TNF
|LTA|LTB|STAT1|CCR5|CXCR3|IL12RB1|IFNGR1|TBX21|STAT4",

            mymeans$interacting_pair,value = T)

th2 <- grep("IL4|IL5|IL25|IL10|IL13|AREG|STAT6|GATA3|IL4R",

            mymeans$interacting_pair,value = T)

th17 <-
grep("IL21|IL22|IL24|IL26|IL17A|IL17A|IL17F|IL17RA|IL10|RORC|RORA|STAT3|
CCR4|CCR6|IL23RA|TGFB",

             mymeans$interacting_pair,value = T)

treg <- grep("IL35|IL10|FOXP3|IL2RA|TGFB", mymeans$interacting_pair,value = T)

costimulatory <-
grep("CD86|CD80|CD48|LILRB2|LILRB4|TNF|CD2|ICAM|SLAM|LT[AB]|NECTIN
2|CD40|CD70|CD27|CD28|CD58|TSLP|PVR|CD44|CD55|CD[1-9]",

                  mymeans$interacting_pair,value = T)

coinhibitory <-
grep("SIRP|CD47|ICOS|TIGIT|CTLA4|PDCD1|CD274|LAG3|HAVCR|VSIR",

                 mymeans$interacting_pair,value = T)

niche <- grep("CSF", mymeans$interacting_pair,value = T)



mymeans %>% dplyr::filter(interacting_pair %in% costimulatory)%>%
```

```
dplyr::select("interacting_pair",starts_with("Osteoblastic"),ends_with("Osteoblastic"))
%>%
  reshape2::melt() -> meansdf


colnames(meansdf)<- c("interacting_pair","CC","means")


mypvals %>% dplyr::filter(interacting_pair %in% costimulatory)%>%

dplyr::select("interacting_pair",starts_with("Osteoblastic"),ends_with("Osteoblastic"))
%>%
   reshape2::melt()-> pvalsdf


colnames(pvalsdf)<- c("interacting_pair","CC","pvalues")
pvalsdf$joinlab<- paste0(pvalsdf$interacting_pair,"_",pvalsdf$CC)
meansdf$joinlab<- paste0(meansdf$interacting_pair,"_",meansdf$CC)
pldf <- merge(pvalsdf,meansdf,by = "joinlab")


summary((filter(pldf,means >1))$means)
pdf("Osteoblastic.costimulatory.pdf",width=9)
pldf%>% filter(means >1) %>%
  ggplot(aes(CC.x,interacting_pair.x) )+
  geom_point(aes(color=means,size=-log10(pvalues+0.0001)) ) +
  scale_size_continuous(range = c(2,4))+
  scale_color_gradient2(high="red",mid = "blue",low ="yellow",midpoint = 1  )+
theme_bw()+
  theme(axis.text.x = element_text(angle = 45,hjust = 1,vjust = 1))
dev.off()
```
